# Calcinosis Cutis in a Seven-Year-Old Male With Hepatic Focal Nodular Hyperplasia and Congenital Partial Venous Drainage

**DOI:** 10.7759/cureus.94078

**Published:** 2025-10-07

**Authors:** Ali Alroumi, Humoud Al-Sabah

**Affiliations:** 1 Dermatopathology, As'ad K Al-Hamad Dermatological Center, Kuwait, KWT

**Keywords:** calcinosis cutis, calcium salts, nodules, pediatrics, skin biopsy

## Abstract

Calcinosis cutis, defined as the deposition of insoluble calcium salts in the skin and subcutaneous tissue, is an uncommon condition in pediatric populations. It may arise from various underlying causes, including metabolic, inflammatory, or structural anomalies, and is classified into several subtypes, each requiring a distinct approach to diagnosis and management. We present the case of a seven-year-old Indian male with a history of massive hepatic focal nodular hyperplasia and a congenital partial venous drainage anomaly, who developed widespread, firm, non-tender, skin-colored nodules over a two-year period. The lesions were distributed across multiple areas, including behind the ears, neck, trunk, inguinal region, and extremities. This case highlights the importance of recognizing calcinosis cutis as a potential dermatologic manifestation in children with underlying systemic abnormalities and the need for individualized evaluation and multidisciplinary management.

## Introduction

Calcinosis cutis is a condition characterized by the abnormal deposition of insoluble calcium salts in the skin and subcutaneous tissue. It is generally uncommon and particularly rare among pediatric patients, especially when presenting with widespread distribution. The condition can be broadly classified into five subtypes based on etiology: dystrophic, metastatic, idiopathic, iatrogenic, and calciphylaxis [1]. Dystrophic calcification is the most common subtype and occurs in areas of prior tissue damage or inflammation despite normal serum calcium and phosphate levels, while metastatic calcification results from systemic mineral imbalance. Other forms are less common and may involve complex systemic or local factors. Although calcinosis cutis is more frequently associated with connective tissue diseases such as dermatomyositis and systemic sclerosis, it can also result from trauma, infections, or congenital vascular abnormalities. Pediatric cases, when they occur, often pose a diagnostic challenge due to overlapping features with other soft tissue disorders and the rarity of underlying systemic associations at a young age [2,3]. This case report presents a seven-year-old male with widespread calcinosis cutis in the context of known hepatic and vascular anomalies. It aims to provide a detailed account of the patient’s clinical presentation, laboratory investigations, imaging studies, and histopathological findings, while discussing the potential underlying mechanisms and relevant differential diagnoses.

## Case presentation

**Figure 1:**
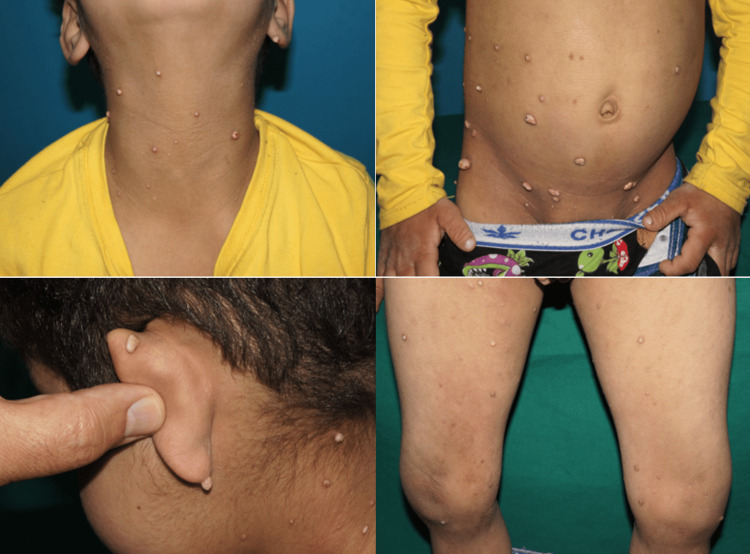
Widespread skin-colored nodules, most noticeably behind the ears and over the neck, trunk, inguinal region, and extremities.

A seven-year-old Indian boy, with a known case of massive hepatic focal nodular hyperplasia and congenital partial venous drainage anomaly since the age of three, presented with a two-year history of multiple nodules throughout his body back in November 2024. The nodules were skin-colored, with a hyperpigmented base and a whitish center, but non-erythematous. They were firm and painless, with varying sizes ranging from around 2 mm to 10 mm. These lesions were disseminated throughout the body, particularly behind the ears and over the neck, trunk, inguinal region, and extremities, sparing the face, palms, and soles, and there was no hair or nail involvement (Figure 1). Otherwise, the patient’s past medical, surgical, drug, and allergy histories were unremarkable, and there was no family history of such lesions or any cutaneous diseases.

**Figure 2:**
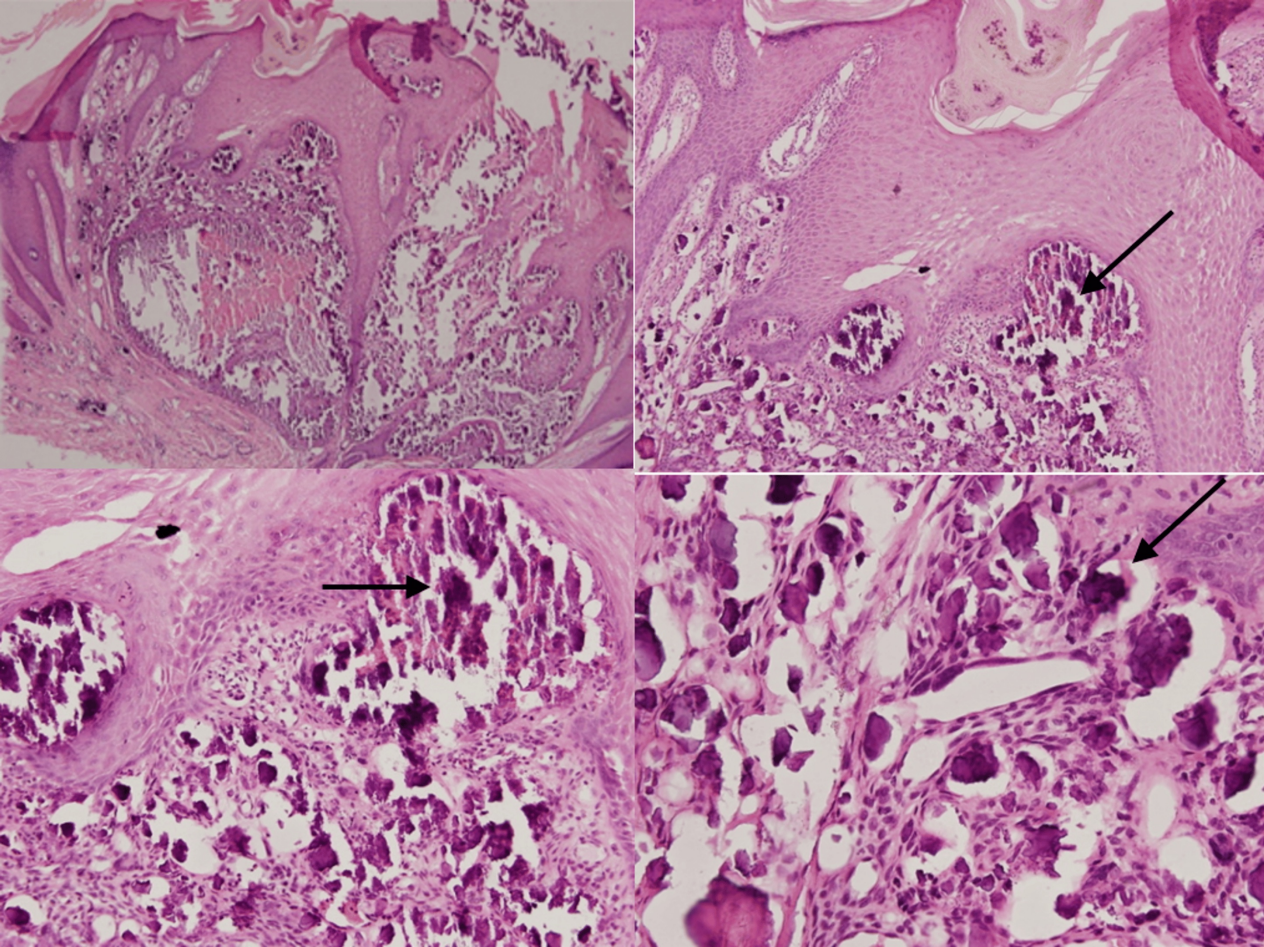
H&E stain showing irregular and deep basophilic deposits with surrounding inflammatory cells

**Figure 3:**
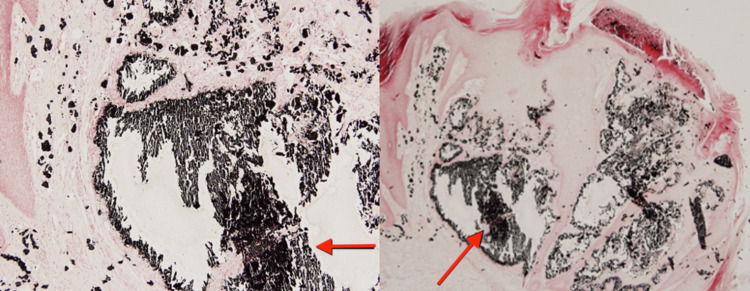
Von Kossa stain showing black deposits depicting calcium salts

Laboratory tests were conducted, including a complete blood count, serum calcium, phosphate, parathyroid hormone, vitamin D, alkaline phosphatase levels, renal function tests, liver function tests, thyroid function tests, and inflammatory and connective tissue disease markers, all of which were within normal ranges. A biopsy was taken from one of the nodules. Histopathological examination with hematoxylin and eosin (H&E) stain showed irregular basophilic deposits within the dermis and subcutaneous tissue with surrounding inflammatory cell infiltrates, which consisted of lymphocytes and macrophages (Figure 2). Von Kossa staining was also applied, showing black staining on the deposits, which indicates calcium salts (Figure 3).

Given that the patient was asymptomatic, with the nodules neither impairing function nor causing pain, the patient underwent surgical excision, but it was done at another center, and no pharmacological intervention was initiated. The management plan focused on clinical observation with periodic follow-up to monitor for disease progression, changes in lesion characteristics, or the development of complications. A multidisciplinary approach was adopted, involving dermatology, pediatrics, and hepatology teams to ensure comprehensive care in view of the patient’s hepatic comorbidity. The family was counseled on the benign course of the condition, potential treatment options, and the importance of regular monitoring. Follow-up visits were scheduled every six months to assess for new lesions, progression of existing nodules, or onset of symptoms.

## Discussion

Calcinosis cutis is a condition characterized by the deposition of insoluble calcium salts in the skin and subcutaneous tissue. According to etiology and serum calcium and phosphate levels, it may be classified as either dystrophic, metastatic, iatrogenic, or idiopathic. Dystrophic calcinosis cutis occurs secondary to local tissue damage in the context of normal calcium and phosphate levels, such as in trauma, infections, or various connective tissue diseases like systemic lupus erythematosus (SLE), systemic sclerosis, dermatomyositis, or panniculitis. Metastatic calcinosis cutis occurs secondary to hypercalcemia with or without associated hyperphosphatemia, such as in chronic renal failure, hypervitaminosis D, hyperparathyroidism, or certain neoplasms like multiple myeloma or adult T-cell leukemias and lymphomas. Iatrogenic calcinosis cutis results from parenteral administration of calcium or phosphate, application of calcium-containing materials in the setting of diagnostic tests like ECG, EEG, or electromyography (EMG), as well as predisposing conditions like tumor lysis syndrome. Idiopathic calcinosis cutis presents in the absence of any underlying tissue disease [1-5]. 

While the exact pathogenesis remains unclear, it is thought to involve several mechanisms. Chronic inflammation may predispose to cascades such as interleukin and tumor necrosis factor activation, while vascular hypoxia may lead to increased hypoxia-associated glucose transporter 1 and vascular endothelial growth factor expression [4]. Ultimately, these factors result in increased calcium deposition and precipitation anywhere in the body, including the extremities, elbows and knees, buttocks, face, and trunk [2]. These deposits present as firm, whitish or yellowish nodules or papules, and most cases are asymptomatic. However, some may present with ulceration, tenderness, movement restriction, and gangrene in severe cases [1,3].

Although rare, calcinosis cutis should remain in the differential diagnosis of any patient with such a presentation. A thorough and detailed history may provide insight into any underlying disease or trauma, and an extensive physical examination may help in ruling out other conditions or in evaluating the severity of the patient’s complaint. Laboratory tests, such as serum calcium and phosphate levels, renal function tests, inflammatory marker levels, and an autoimmune panel, are also necessary to determine any existing disorders [1,2]. Investigating serum calcium and phosphate levels in particular can help distinguish between certain types of calcinosis cutis, with hypercalcemia and/or hyperphosphatemia indicating metastatic, and a normal calcium and/or phosphate level indicating otherwise [2]. Imaging with plain film X-ray or CT may also be of use to differentiate calcified nodules from other masses and to evaluate the extent of the disease [1,3]. Nonetheless, the gold standard remains a skin biopsy and staining with H&E and Von Kossa, showing basophilic deposits, inflammatory cell infiltrates, and calcium deposits [2,6]. 

Given the patient’s normal laboratory results, absence of systemic metabolic abnormalities, and prior history of hepatic and vascular anomalies, this case is most consistent with the dystrophic type. Iatrogenic and idiopathic causes were considered but excluded due to lack of prior local trauma, medication use, or unexplained etiology. We further hypothesize that altered hemodynamics due to hepatic focal nodular hyperplasia and congenital partial venous drainage anomaly may have contributed to local tissue hypoxia, creating a milieu favoring dystrophic calcification. The interplay between vascular stasis, impaired clearance of metabolic by-products, and chronic low-grade inflammation may explain the localized calcium deposition in the absence of systemic abnormalities, particularly in pediatric patients.

The pharmacological approach to this condition aims to relieve symptoms, improve function, and prevent complications. Medications include calcium channel blockers like diltiazem, bisphosphonates like alendronate, sodium thiosulfate, or intralesional corticosteroids, with the use of immunosuppressants reserved for cases with underlying autoimmune diseases. The non-pharmacological approach revolves around surgical or carbon dioxide removal of the lesions, especially in symptomatic cases, and remains the mainstay of treating this disease [6,7]. Other options may include minimally invasive therapies like laser and extracorporeal shock wave lithotripsy, which, in limited reports, have shown capacity for partial or complete remission of microcalcifications and pain reduction, albeit with risk of cutaneous side effects such as scarring and hyperkeratosis [8]. As the patient was asymptomatic and the nodules did not impair function, only surgical excision was needed without the need for pharmacological intervention. The patient was placed under clinical observation with periodic follow-up to monitor for progression. A multidisciplinary approach involving dermatology, pediatrics, and hepatology was discussed to ensure comprehensive care.

Although the approach for each case of calcinosis cutis may vary according to the patient's presentation, the most highly recommended course of action remains excision, with many documented cases reporting no recurrence [9]. Moreover, conservative approaches aimed at increasing blood flow to affected extremities may help facilitate treatment, such as smoking, cold, and stress avoidance [10].

## Conclusions

Recognizing any potential cause of calcinosis cutis is essential for its management and treatment. However, management will vary from patient to patient, as it should be tailored per the individual while taking into consideration other diseases they may have. In pediatric cases, early identification of systemic or metabolic abnormalities can be particularly important in preventing progression. In addition to addressing the underlying etiology, symptomatic treatment and multidisciplinary follow-up may be necessary for optimal outcomes. Further studies and case reports are needed to better characterize rare presentations of this condition and guide evidence-based therapeutic strategies.
